# Psychological and behavioral approaches to cardiac patients facing specific adjustment challenges

**DOI:** 10.3389/fpsyg.2012.00476

**Published:** 2012-11-01

**Authors:** Gian M. Manzoni, Giada Pietrabissa, Gianluca Castelnuovo

**Affiliations:** ^1^Psychology Research Laboratory, Istituto Auxologico Italiano IRCCS, Ospedale San GiuseppeVerbania, Italy; ^2^Department of Psychology, Catholic University of MilanMilan, Italy

To some extent, all cardiac patients are challenged by their heart disease. Challenging issues concern the adjustment of patients to the cardiovascular disease and involve the physical, psychological, behavioral, and social dimensions of the person. Clearly, heart disease, its physical impact and the clinical sequelae (treatment and rehabilitation procedures) challenge patients at multiple levels by critically upsetting the homeostasis of their bio-psycho-social systems. Challenges may be very easy as well as very hard to accomplish and this variability does not depend only on the seriousness of the cardiac illness or on the impact of the correlated clinical and rehabilitation procedures, but it is also related to the psychosocial characteristics of patients, including personality and coping styles. When patients have difficulties in facing the multi-dimensional challenges of heart disease, they may show high levels of stress, anxiety, and depression as well as problematic behaviors such as resistance and poor adherence to treatment and preventive prescriptions, including behavioral modifications such as smoking cessation and physical exercise improvement.

Given the huge amount of empirical evidence supporting the fact that smoking, physical inactivity, unhealthy diet, harmful use of alcohol as well as psychological distress are modifiable risk factors for cardiovascular morbidity and mortality, patients showing difficulties in adjusting to cardiac illness and the correlated clinical demands should be detected and then helped with behavioral and psychological interventions in facing the multi-dimensional challenges they are not able to overcome themselves.

This book, edited by an experienced health psychologist in the field of “behavioral cardiology” and written by an international group of outstanding researchers and clinicians working in the expanding area of bio-psycho-social approaches to cardiac patients, covers firstly the psychological challenges and management needs of patients affected by specific cardiovascular conditions. With few exceptions, all chapters in the first part deserve particular consideration for their specialty contents. Clinicians working with cardiac patients affected by specific cardiovascular conditions such as ICD implantation for ventricular fibrillation, atrial fibrillation, heart failure, Takotsubo syndrome, and cardiac transplantation, may find in them extensive information about the unique psychological challenges and difficulties of living with such conditions and they may also acquire specialty knowledge on tailored psychological approaches to specific needs management.

In the second and last part of the book, various psychological and behavioral interventions for cardiac patients are described. With the exception of the review chapter about anxiety and depression as risk factors for cardiovascular disease, whose inclusion in this section is questionable, the great part of issues that are covered along the second half of this book were rarely or never addressed in other similar publications. Bright spots are the chapters on computerized therapies and on the management of sleep problems, while a missing issue is the psychological and behavioral management of obese patients with heart disease, for which tailored multi-disciplinary interventions have already been planned and assessed (Manzoni et al., 2010, 2011).

The declared purpose of this book is to inform clinicians who practice on the front line of the psychological and behavioral management of cardiac patients on topics where the research findings have not yet caught with practice and, at the same time, to provide them concrete, immediately applicable, translation of clinical research findings into tailored practical approaches (Castelnuovo, 2010a,b). It is noteworthy that clinicians whose this book is addressed will find in it all that was promised.
